# Miniature low-cost γ-radiation sensor for localization of radioactively marked lymph nodes

**DOI:** 10.1177/09544119211058918

**Published:** 2021-12-01

**Authors:** Merlin Behling, Felix Wezel, Peter P Pott

**Affiliations:** 1 Institute of Medical Device Technology, University of Stuttgart, Stuttgart, Germany; 2 Department of Urology and Pediatric Urology, University of Ulm, Ulm, Germany

**Keywords:** Experimental evaluation, gamma detector, low-cost, miniaturization, radioguided surgery

## Abstract

Detection of metastasis spread at an early stage of disease in lymph nodes can be achieved by imaging techniques, such as PET and fluoride-marked tumor cells. Intraoperative detection of small metastasis can be problematic especially in minimally invasive surgical settings. A γ-radiation sensor can be inserted in the situs to facilitate intraoperative localization of the lymph nodes. In the minimally invasive setting, the sensor must fit through the trocar and for robot-aided interventions, a small, capsule-like device is favorable. Size reduction could be achieved by using only a few simple electronic parts packed in a single-use sensor-head also leading to a low-cost device. This paper first describes the selection of an appropriate low-cost diode, which is placed in a sensor head (Ø 12 mm) and characterized in a validation experiment. Finally, the sensor and its performance during a detection experiment with nine subjects is evaluated. The subjects had to locate a ^137^Cs source (138 kBq activity, 612 keV) below a wooden plate seven times. Time to accomplish this task and error rate were recorded and evaluated. The time needed by the subjects to complete each run was 95 ± 68.1 s for the first trial down to 40 ± 23.9 s for the last. All subjects managed to locate the ^137^Cs source precisely. Further reduction in size and a sterilizable housing are prerequisites for in vitro tests on explanted human lymph nodes and finally in vivo testing.

## Introduction

Metastatic spread is a hallmark of malignant and deadly cancers. Under certain circumstances, metastasis-directed therapy by, that is, surgical resection or radiotherapy may be life prolonging or even curative in oligometastatic disease, when resection of metastatic lesions can be achieved.^1^ Detection of small metastasis at an early stage of disease, that is, in lymph nodes can be achieved by modern imaging techniques, such as positron emission tomography (PET), usually combined with computed tomography (PET/CT) or magnetic resonance imaging (PET/MRI). For diagnostic PET, tumor cells can be marked with fluoride ^18^F or other radiopharmacons attached to ligands that are characteristic for tumor cell metabolism, such as fluorodeoxyglucose (FDG), or ligands that specifically bind to tumor cells, that is, prostate specific membrane antigen (PSMA) to detect prostate cancer cells.^2^ By using such imaging modalities, even small tumor cell clusters can be detected in the human body.^3^

However, intraoperative detection of small tumor lesions can be problematic, especially in the case of small or atypically located lesions, which cannot be visualized. This is further complicated in minimally invasive surgery, where direct manual access is not possible.

In recent years, salvage surgery for oligo-metastatic prostate cancer has come more and more into focus especially due to advances in PSMA-based PET. It was shown that intraoperative detection of cancer cells labeled with radiopharmaceutical 99mTc attached to PSMA by using a γ-radiation sensor was feasible. Moreover, such radioguided surgery could improve the prognosis in some patients. In early clinical experiences, the removal of the lymph node bearing tumor cells was confirmed outside the body.^4^

For prostate cancer, robotic-assisted laparoscopic radical prostatectomy has been established as minimal invasive standard procedure.^5^ In such a minimally invasive setting, a γ-radiation sensor that can be inserted in the situs should make it easier to locate the lymph nodes intraoperatively. In order to achieve this, the sensor must be inserted through a conventional trocar and guided over the tissue surfaces to be examined with the aid of rigid hand-held laparoscopic instruments or end effectors guided by robotic manipulators. This application requires sterilizable devices as well as dimensions that allow insertion through the trocar.

**Figure 1.**
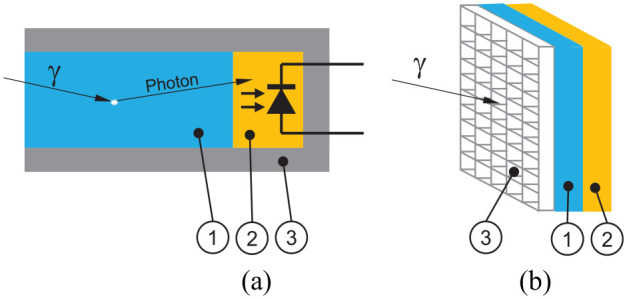
(a) Schematic of a gamma quant detecting probe. (1) Scintillator crystal converting an incoming gamma quant into a photon, (2) photo diode detecting the photon, (3) tungsten housing. (b) Schematic of a gamma camera. The incoming gamma quant passes the tungsten collimator layer (3) and is converted to a photon in the scintillator crystal layer (1) and then finally detected by one of the photo diodes in the detector layer (2) doing so, a spatial encoding of incoming gamma quants is achieved.

The state of the art comprises handheld probes (Figure 1(a)) and gamma cameras (Figure 1(b)). Such a probe can consist of a handle and a shaft,^6,7^ which is guided through the trocar. The sensitive sensor surface is located at the end of the shaft. The directional sensitivity for gamma quants is achieved by using apertures, for example, made of tungsten, which provide a relatively good shielding. In the standard setting, the actual sensor consists of a thallium-doped sodium iodide – NaI(Tl) scintillation crystal, whose photon emission (wavelength range 325–550 nm, maximum emission at 415 nm) is read out by a positive intrinsic negative (PIN) photodiode. The probe acts as a single-point detector and delivers no information on spatial distribution of γ-radiation. According to the manufacturer’s specifications,^6^ the sensitivity is 23 kcount/s/MBq in a gamma energy range from 60 to 511 keV. The background noise is 0.3 counts/s.

Gamma cameras (Figure 1(b)) consist of a flat detector array of scintillation crystals and photodiodes and a collimator arranged in front of it. Geometry and material are matched to the isotope to be detected. The resulting image (“scintigraphy”) encodes the local distribution of the radionuclide, for example, in an organ. Such systems have been available for many years as permanent installations^8^ and as hand-held devices.^9,10^ In contrast to an optical camera there is no lens focusing an image, thus the sensing area must be brought to the organ as close as possible.

The described devices are not suitable to be used by minimally invasive robotic surgical systems as their size does not allow robotic manipulation. It is thus necessary to provide a small, capsule-like device that fits through the trocar and can be navigated while being gripped by the robot’s end effector, which is important for probe positioning.^11^ The development of a reusable drop-in gamma probe for robotic manipulation has been recently described by Dell’Oglio et al.^12^ Size reduction could be achieved by using only a few simple electronic parts compiled in a single-use sensor head.

This paper first describes the selection of an appropriate diode. Requirements of a suitable diode are high sensitivity (pulses per time interval at a given radiation density), high signal strength for each pulse – even without the implementation of a scintillator crystal, low dark current at 37°C, low capacity, small outlines, and low price to allow compact and low-cost devices. Secondly, during a validation experiment, the selected diode is characterized considering temperature, angle between diode and radiation source, and the influence of the distance between radiation source and diode. Thirdly, the first prototype of such a sensor and its performance during a detection experiment is presented. Here, the hypothesis is that the frequency of the occurrence of a sound impulse is a measure for the local γ-radiation intensity and thus enables the surgeon to locate a lymph node labeled by a radioactive tracer in an (in vitro mimicked) site using the presented sensor.

## System design

To achieve the goal of small outlines, a detecting principle using PIN photo diodes was chosen.^13^ A PIN photodiode is similar to a regular (PN) photo diode, but with an intrinsic layer between the p-doped and n-doped layers. This layer is intrinsically conductive, and consists of pure or only very lightly doped silicon. The advantage of this additional intermediate layer becomes apparent when a reverse voltage is applied. The depletion region becomes significantly larger compared to a regular diode. This has a positive effect on the detection probability and also reduces the capacity of the diode, which limits the detection frequency. In case a gamma quant interacts with the material in this region, a pair of charge carriers is generated, which again result in a current pulse. This pulse can be conditioned by an amplifier circuit. It has to be considered, that a PIN photo diode is basically detecting light. For the intended purpose it has thus to be covered by an opaque layer. As it is also detecting α–and β–radiation it has to be shielded against this by a layer of steel.

**Figure 2.**
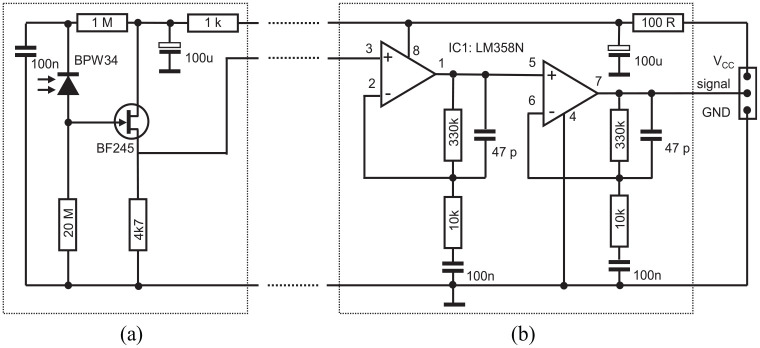
Basic amplifier circuit. Primary stage of the amplifier circuit located directly near to the sensor photo diode (left): (a) primary amplifier stage. Main amplifying circuit located next to the electronics for evaluating the signal (right): (b) secondary amplifier stage. The dashed lines indicate the cable between sensor head and main amplifier circuit.

The amplifier circuit has two stages to improve the signal-to-noise ratio (SNR) and at the same time to realize the smallest possible measuring head. The first stage located directly next to the PIN diode amplifies the current pulse after gamma quant detection to a millivolts-level voltage that can be sent to the next stage without too much losses. This also improves SNR. The second stage then amplifies the signal to a level that can be read out by the micro controller’s analog input (∼3 V). The principle circuit is shown in Figure 2.

The sensor head is connected to the extracorporeally arranged control unit by means of a tension-proof robust shielded cable. The sensor housing can be held via the cable and manipulated by any surgical or endoscopic grasping forceps in the situs. This is to ensure easy navigation of the sensor head while keeping the outlines as small as possible. With the help of the cable the sensor head can be retrieved from the situs at any time.

**Figure 3.**
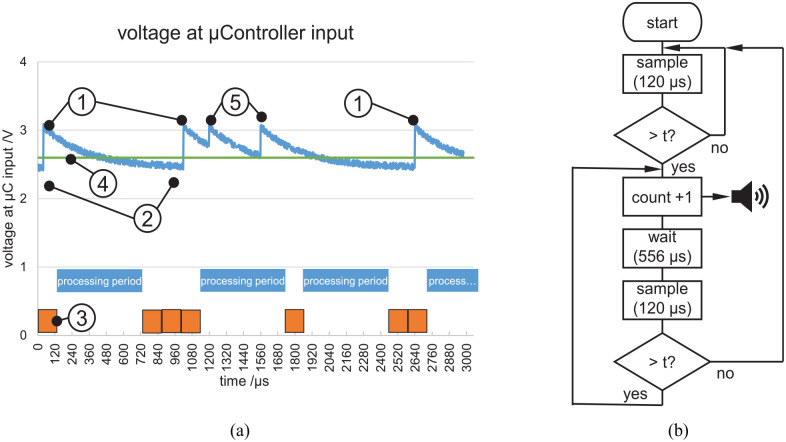
(a) Exemplary signal at the microcontroller input. (1) Amplified pulse after gamma quant detection, (2) voltage derived from dark current of the photo diode, (3) one sampling period (120 µs), (4) threshold for pulse detection, (5) multiple pulses within one processing period (556 µs). (b) Algorithm running on the microcontroller (“t” abbreviates threshold).

The second stage of the amplifier circuit (Figure 2(b)) is set up from two OP amps with high input impedance. They amplify the voltage of the first stage to be read out by a microcontroller (Arduino™ MEGA). One single sample readout needs 120 µs on average (8.3 kHz sampling rate, see Figure 3(3)). After detection of a pulse (Figure 3(1)) the algorithm waits 556 µs (without sampling) for the signal to decay. If after this time the input voltage is below the threshold (Figure 3(4)) a next pulse can be counted. If during a processing period another pulse is measured, the signal will be above the threshold at the end. In this case a second pulse will be counted and a new processing period is triggered. A third or higher pulse (Figure 3(5)) would be discarded. For a pulse to be counted it has to be 5% above (see Figure 3(4)) dark current level (see Figure 3(2)). The processing period has a duration of 556 µs on average. Thus, a maximum number of $1/\left(556+120\right)\;\cdot \;10^{-6}=1479$ pulses per second can be detected by the microcontroller. Accordingly, the sensor saturates at values above 1479 pulses per second.

For each detected pulse the microcontroller produces a sound of a frequency of 1 kHz lasting approx. 25 ms, which are made audible to the operator via a loudspeaker. The frequency of the sound appearance per time period allows a quantitative assessment of the radiation intensity depending on the location of the measurement. The maximum rate of pulses is $\left(1000\;\mathrm{ms}/s\right)/25\;\mathrm{ms}=40s^{-1}$ (continuous sound).

### Preliminary experiment

**Figure 4.**
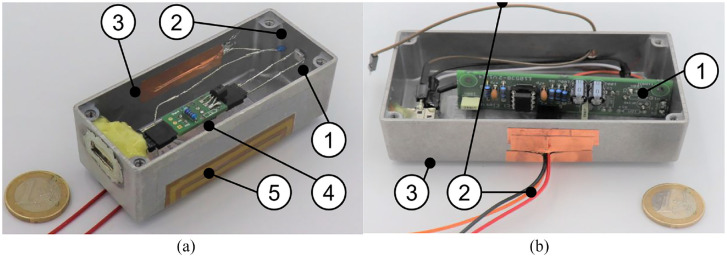
Set up of the electronics for preliminary tests of diodes: (a) shows the prototypic sensor head ((1) diode under test, (2) temperature sensor, (3) shielded housing, (4) primary stage of the amplifier, and (5) electric heating to 37°C). (b) Shows main amplifying circuit ((1) printed circuit board, (2) cables to micro controller, and (3) shielded housing).

Aim of the preliminary experiment was to identify suitable photodiodes for the sensor. A good photodiode produces a large number of clearly detectable pulses at a given rate of gamma quants and specific energy. Dark current and capacity should both be low for clear and fast pulse detection. For the pre-test, the circuit for pre-amplification and the actual sensor are accommodated in a larger aluminum housing (see Figure 4(a)). The sensitive surface of the diodes (Table 1) is arranged perpendicular to the axis of the housing at its distal end. In front of the sensitive surface of the sensor, the aluminum housing (thickness 1 mm) prevents the penetration of α–radiation and blocks large amounts of the β–radiation. It should be noted that the measuring principle requires that radiation of a source behind the sensor surface is also detected.

**Table 1.** Diodes under test. Values apply for capacity and dark current at 20 V reverse voltage.

Type	Manufacturer	Cost (Δ)	Active area (mm^2^)	Capacity (pF)	Dark current (nA)
BPW34	OSRAM GmbH, Munich, GER	1	7.02	12	3.8
S1223-01	Hamamatsu Photonics K.K, Naka-ku, JP	10	13	20	0.2
FDS100A	Thorlabs Inc, Newton, NJ, USA	15	13	24	1
PS13-5B	First Sensor AG, Berlin, GER	35	13	72.5	0.07

### Validation experiment

**Figure 5.**
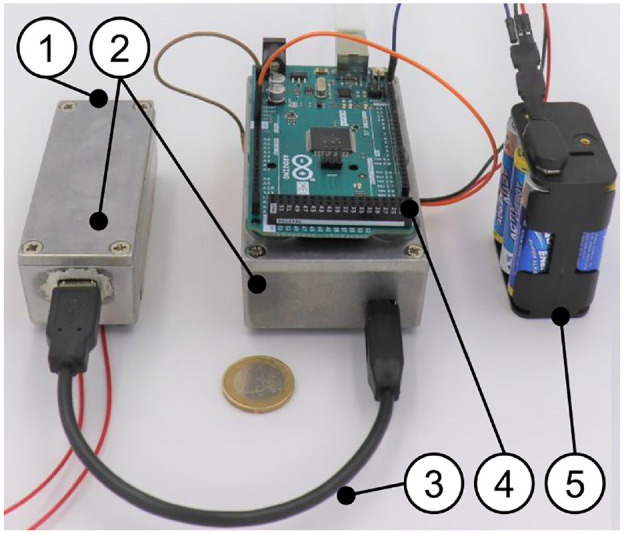
Complete system for preliminary testing of the diodes. (1) Housing end with diode (see Figure 4(a))), (2) shielded housing for amplifying electronics, (3) shielded cable, (4) microcontroller for signal processing, and (5) DC power supply (battery, 9 V). Not shown: heating control circuit and power supply.

Aim of the validation experiment was to prove, that the selected diode (BPW34) is suitable to detect γ-radiation emitted by a ^137^Cs source. To derive an estimate of the sensitivity, temperature dependency of the signal, and the dependency of the signal with regard to the distance and angular displacement of the selected diode, the system shown in Figure 5 was used.

### Detection experiment

**Figure 6.**
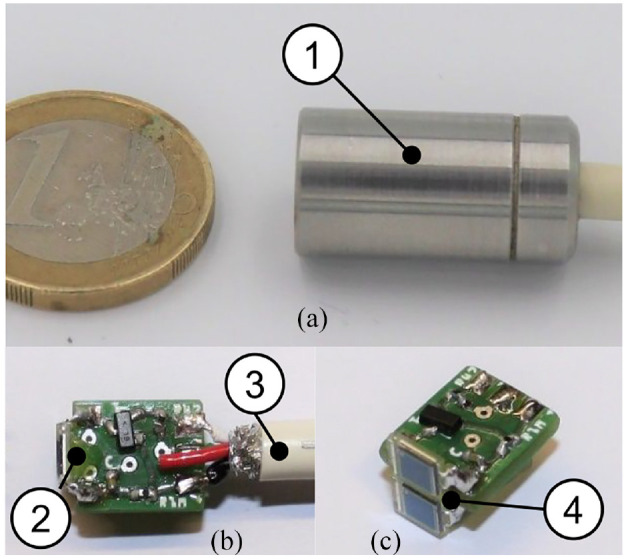
Sensor head including primary stage of the amplifier: (a) Photo of the device (Ø 12 × 25 mm), (b and c) internal view of the circuit. (1) stainless steel housing of the photo diode array and the primary stage of the amplifier circuit, (2) printed circuit of the primary stage of the amplifier circuit, (3) shielded cable, and (4) photo diode array.

The pre-clinical applicable system (see Figure 6) consists of a cylindrical stainless steel tube of 12 mm diameter and 25 mm length, which forms the sensor head (see Figure 6), blocks α– and β–radiation, and allows basic hygiene measures. This contains the actual sensor diodes and the primary stage amplifier for signal pre-amplification (see Figure 4(a)).

## Material and methods

### Preliminary experiment

To derive a test signal, a conventional incandescent gas mantle was used. This contains Thorium(IV)-oxide and therefore through natural decay ^212^Pb, which both emit γ-radiation. ^212^Pb is dominating the spectrum with a gamma quant energy level of 240 keV. The gas mantle was folded to a size of 3×3 cm^2^ and kept in a transparent polyethylene bag.

For the experiment, the plastic bag was first attached directly to the housing leading to a minimum distance of 1 mm between gamma source and detecting diode, and secondly mounted at a distance of 18 mm. The latter is expected to be the practical maximum detection distance during surgery. For each diode and distance, a measuring interval of 1 min was chosen and detection events were counted. Ten consecutive repetitions of the measurements were performed.

As the dark current of the photo diodes is strongly affected by temperature, the primary amplifier housing temperature at the place of the diode was kept at 37°C ± 1°C by a dedicated heating circuit. This circuit was set up from a resistive heating foil (see (5) in Figure 4(b)), a temperature sensor next to the diode (see (2) in Figure 4(a)), and a PID controller implemented on a dedicated second microcontroller (Arduino™ UNO).

### Validation experiment

**Figure 7.**
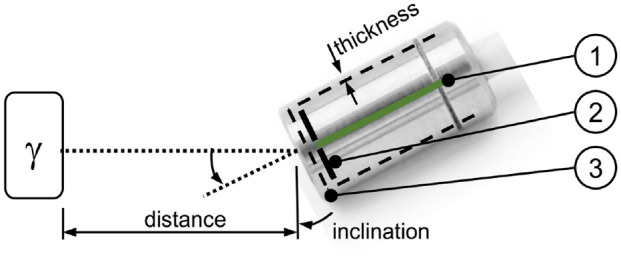
Illustration of the test setup and definition of parameters. (1) PCB of the primary stage of the amplifier, (2) two diodes, and (3) stainless steel casing.

The sensor head ((1) in Figure 5), including a single BPW34 photo diode, was put in front of the plastic bag containing the incandescent gas mantle. First, the temperature dependency of signal strength after the main amplifying circuit and number of pulses was measured at 22°C (room temperature) and 37°C (body temperature) at a distance of 1 mm. Ten measurements of 60 s duration were performed each (Figure 7).

Then the dependency of the inclination angle of the diode with regard to the plastic bag containing the incandescent gas mantle (0°, 45°, 90°) was evaluated at a distance of 1 mm at room temperature. Ten measurements of 60 s duration were performed each. Again, the number of pulses per minute and signal strength were assessed.

Finally, the number of detected events per minute was assessed using a ^137^Cs source (Amersham Buchler, activity: 370 kBq in 1977, today approx. 138 kBq, energy: 612 keV, active area: approx. 4 mm^2^) at distances of 1, 2.5, 5, 10, 20, and 40 mm. For each distance, during 20 min at room temperature, the number of pulses was measured, and the average was calculated.

### Detection experiment

**Figure 8.**
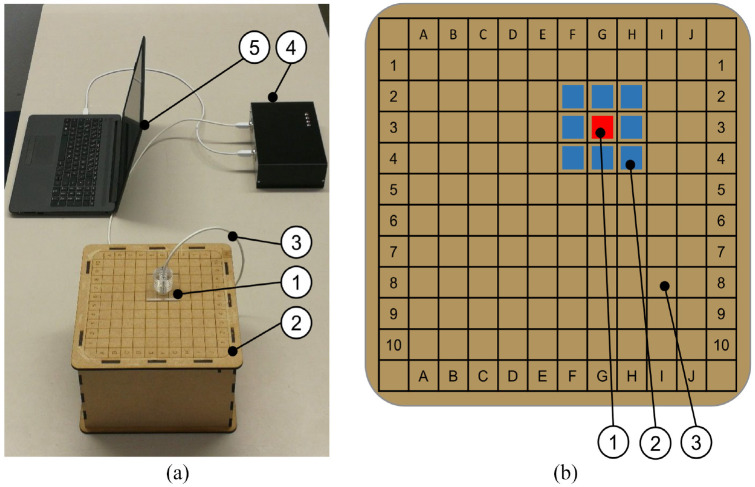
(a) Setting for the detection experiment. (1) Sensor head including primary stage of the amplifier fixed in acrylic glass support, (2) top plate of the box containing the ^137^Cs γ-radiation source, (3) shielded cable), (4) housing for signal conditioning electronics, (5) computer for data processing. (b) Illustration of the top plate surface. (1) Exemplary position of the radiation source (not visible to the subjects), (2) neighboring field, (3) any other field).

For the preclinical validation trial, the structure described below was chosen. The experiments were carried out in the laboratory with nine volunteers (4 f, 5 m, Ø age 25 ± 2.5 years) who had no previous medical training. The subjects had to unveil the location of the ^137^Cs source hidden in a wooden box (made from medium-density fiber board, Figure 8(a))). The time taken to find the sample and the localization accuracy were recorded.

A square base area was divided into 10 columns (A–J) and 10 rows (1–10) (see Figure 8(b)). Each square had a size of 20 × 20 mm^2^ and was formed by engraved lines. The radiation source was placed underneath the plate in such a way that the active area was always centered on one field. The effective distance between the radioactive sample of ^137^Cs was thus the thickness of the plate (5 mm). The grid and the names of the columns and rows were visible to the test persons.

For the actual experiment, the radiation source was randomly positioned under a field of the cover plate without this being directly visible to the subjects. The subjects held the sensor in a support (made from acrylic glass) that forces the sensor to be placed perpendicular on the plate and the frontal surface of the sensor head touched the cover plate. As a consequence, the same minimum distance of 5 mm between radiation source and sensor head and thus a maximum signal was always achieved. The subjects were then asked to locate the radiation source as precisely as possible starting with the sensor support directly on top of the cover plate. They were allowed start from an arbitrary starting point deploying a self-selected search strategy. The acoustic signal triggered by each detection of a gamma quant was always audible to the subjects. The shorter time between the acoustic pulses, the nearer the sensor was to the radiation source.

The time between the start of the search and the statement that the sample was found was measured by the test director. Whether the measurement was correct was also recorded and communicated to the test persons after the last trial. The test was repeated seven times per subject. To assess the localization quality, it was recorded for each trial whether the sample was found exactly (“A”), a neighboring field was determined (“B”), or a field even further away was determined by the subjects (“C”). See Figure 8(b) for details.

### Statistics

**Figure 9.**
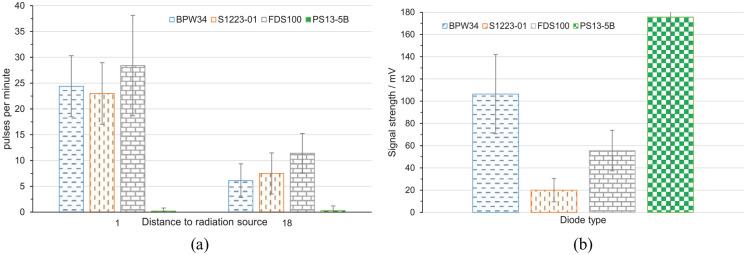
Results of the preliminary experiments for choosing the diode: (a) detected pulses per minute at 1 and 18 mm distance to radiation source. (b) Signal strength of the four diodes tested while detecting a gamma quant.

To evaluate the preliminary tests, 10 periods of 60 s were assessed. The number of pulses per measurement period was then averaged and transferred to Figure 9 as a function of distance.

For the validation experiment averages of the measured values were calculated. Also, a Student’s *t*-test was performed to assess significance levels. *P* values less than 0.05 were considered statistically significant.

To evaluate the detection experiment, a learning curve of the required time was plotted over all test persons. The quality of the detection tests would be recorded for each test person.

## Results

### Preliminary experiment

The preliminary experiment allowed to choose the right diode for further studies. Figure 9(a)) shows the number of pulses per minute for the diodes listed in Table 1. The diode FDS100 has highest value for pulses per minute (sensitivity) either at 1 mm distance or 18 mm. Figure 9(b) depicts the signal strength of the assessed diodes. Diode PS13-5B has the highest output signal in case of detection. However, it detects less than one event per minute.

Following these results and the information collected in Table 1, the diode BPW34 was chosen as it delivers a practical number of pulses per second and a good signal strength at lowest cost, smallest outline, lowest capacity, and a reasonable dark current.

### Validation experiment

**Figure 10.**
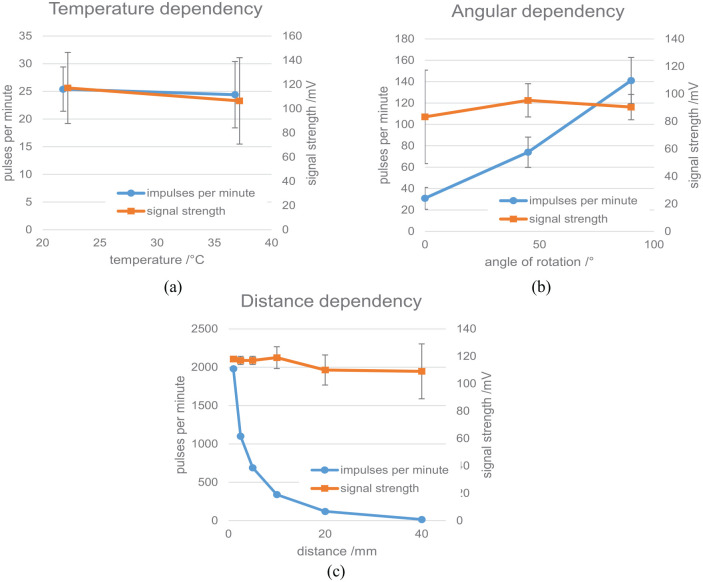
Results of the validation experiment with a single BPW34 photo diode: (a) temperature dependency (note that for better visibility the display of the values has been slightly shifted horizontally), (b) angular dependency, and (c) distance dependency.

During the validation experiment the dependency of signal strength and the number of detected pulses per minute with regard to temperature, angular displacement, and distance were assessed. A visualization of the results is given in Figure 10.

Between room temperature and body temperature the number of detected pulses by the photo diode is reduced by 6% (*p* = 0.7; not significant).

Rotating the photo diode led to a pronounced reduction in detected pulses while the signal strength was not affected significantly. From the highest value of 140 ± 22 pulses per minute at 90°, roughly half of that is achieved at 45° and 30 ± 10 pulses were detected with the diode’s sensitive plane in parallel with the radiation axis. While the number of detected pulses changed significantly (*p* < 0.001), the signal strength did not change significantly for 0° versus 45° (*p* = 0.064) and 0° versus 90° (*p* = 0.25) but the difference between 45° and 90° inclination was significant (*p* = 0.042).

Altering the distance between the ^137^Cs source and the photo diode strongly affected the number of detected pulses while the signal strength again was not affected significantly.

The ^137^Cs source emits 138,000 gamma quants per second evenly distributed in all spatial directions. Given the activity of the source, the number of events on the surface of a sphere of radius *r* is

$$L=\frac{\mathrm{Acivity}}{A_{\mathrm{sphere}}}=\frac{138\mathrm{kBq}}{4\pi {\left(5\;\mathrm{mm}\right)}^{2}}=439\frac{\mathrm{events}}{mm^{2}\;\cdot \;s}$$

At a distance of 5 mm a number of 690 counts per minute could be detected by the selected diode. Thus, for the sensor area of 7.04 mm^2^ (single diode, see Figure 4) a maximum of 185,434 pulses per minute could be expected at 100% sensitivity. The measured rate of 690 pulses per minute leads to a sensitivity of 0.37%.

### Detection experiment

**Figure 11.**
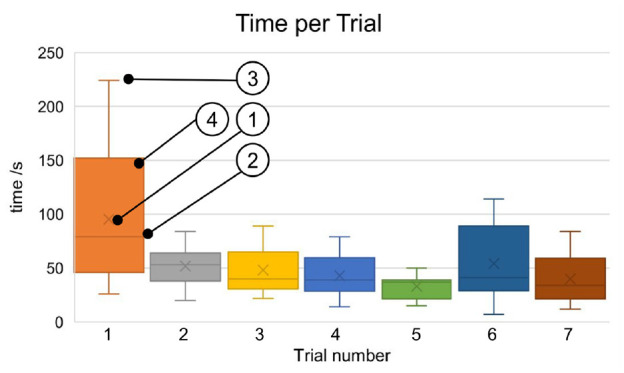
Time needed for each trial over all subjects. (1) Average value, (2) median (3) extreme values, and (4) box depicts 25–75 percentile.

The time needed by the subjects to complete each run was averaged for each trial and is shown in Figure 11. This shows a learning curve with a mean value of 95 ± 68.1 s for the first trial up to 40 ± 23.9 s for the last trial. However, this appears not to be significant.

Finally, the evaluation of the error rate showed that in all cases the test persons were able to determine the position of the source correctly.

## Discussion

The use of current rigid laparoscopic gamma probes can be problematic in robot-assisted procedures because the rigid devices prove limited orientation of the probe head. The aim of our work thus was trifold:

1. During a dedicated preliminary experiment using a low-dose γ-radiation source, the feasibility of setting up a low-cost gamma detector for medical purposes from simple off-the-shelf parts and lab equipment was assessed with the aim of selecting an appropriate and cheap diode (BPW34). The selected diode has a bulk cost of less than 1 € allowing cost-effective realization of such a device. Even a disposable sensor could be envisioned, which would be economically more attractive compared to expensive reusable devices, including costs for re-sterilization.
2. In the validation experiment two BPW34 diodes were mounted into a stainless steel cover to enhance sensitivity. These were then assessed with regard to temperature dependency and the influence of angular and spatial displacement. It became clear that at body temperature the sensitivity is slightly reduced as the influence of dark current rises with temperature. Still, the results show, that the threshold of the detection algorithm is set well. Rotating the diode out of the perpendicular plane led to a pronounced reduction of the detected pulses. This is due to the fact that the projected area of the sensitive plane of the diode penetrated by the gamma quants becomes smaller. Because of the plane having thickness larger than zero, the gamma quants have to come through a thicker layer thus increasing the probability of detection. This can explain why at 0° still a reasonable number of pulses was detected. Finally, increasing the distance between the source and diode led to a reduction of the number of pulses detected. Looking at the inverse square law, an even higher reduction could have been expected. However, this law applies only for point-sized sources. The ^137^Cs source deployed in this study has a size of 4 mm^2^ while the active area of the photo diode has a size of 7.02 mm^2^. Thus, this law can be used as an estimate only.
  The sensitivity of the device is rather low compared to commercial devices (e.g. Chrystal Photonics and Berlin^6^). This can be explained by the fact that the sensor presented does not rely on a scintillator crystal. Also, the microcontroller is limiting the number of counts per second to its loop execution speed – reducing measurement dynamics. While the signal is processed, no new events can be counted. Lastly, the circuit also needs some microseconds to recharge the capacity of the photo diode. However, the use of a simple PIN diode greatly reduces cost and still allows for a sufficient sensitivity and the recharge time is far shorter than the time needed for the read-out by the microcontroller. The acoustic display of the number of detected pulses is limited to 40 per second. Whether this is sufficient to detect lymph nodes in situ has to be assessed in a future pre-clinical experiment.
3. In the third experiment the quality and time needed detecting a γ-radiation source by human subjects was assessed. The chosen radiation source consists of ^137^Cs with an activity of 138 kBq. Due to the short half-life of ^18^F it was not possible to use this material in the setting. However, the activity of the ^137^Cs source is comparable to human lymph nodes during surgery after PET imaging, which is between 1 kBq and 1 MBq.^14^ The γ-radiation source was hidden underneath a 5 mm strong medium-density fiberboard. γ-radiation travels through this material with almost no attenuation. This can be compared to the surgical situation where the layer between sensor head and radiating tissue would mainly consist out of water. It became clear that the selected special resolution of the experimental setup (Figure 8) was too coarse – compared to the clinical use case – to derive a meaningful result of spatial sensitivity. Future pre-clinical experiments will take this into account to come closer to the anatomical specifications. The subjects’ detection speed showed a slight learning curve for the time needed to detect the position of the source. This allows the conclusion that the acoustic user interface allows intuitive handling of the system.

The experiments showed principal suitability of the BPW34 diode to detect a ^137^Cs γ-radiation source. The applicability of the sensor in the medical field must be further assessed. Future developments will comprise a reduction in size of the device, the usage of a hygienic housing of the diode and primary stage of the amplifier to allow in vitro tests on explanted human lymph nodes and finally in vivo testing. Also, a comparison to state-of-the-art devices available in the operating room will be performed.
